# Supplementary material for: Ca2+ oscillation in vascular smooth muscle cells control myogenic spontaneous vasomotion and counteract post-ischemic no-reflow
Source: Commun Biol. 2024 Mar 15;7:332. doi: 10.1038/s42003-024-06010-1 (PMC10942987; doi:10.1038/s42003-024-06010-1)
Supplement: Supplementary file 3 — Description of Additional Supplementary Files [file 42003_2024_6010_MOESM3_ESM.pdf]

Description of Additional Supplementary Files

File name: Supplementary data 1

Description: All the source data behind the graphs in the paper

File name: Supplementary movie 1

Description: 2PLSM time-lapse imaging of the cerebral pial vessels, including the arteriole, venule, and penetrating arteriole (PA) in SMACreER:Ai47 mice injected with RhoB intravenously under 2PLSM.

File name: Supplementary movie 2

Description: Time-lapse imaging of MCA before and after (occ.2hrep.22h) ischemic stroke in SMACreER:Ai47 mouse under 2PLSM. Coupled with the real-time radius changes trace of arteriole before (dash line) and after (solid line) (occ.2hrep.22h) ischemic stroke.

File name: Supplementary movie 3

Description: Time-lapse imaging of MCA before and after (occ.2hrep.22h) ischemic stroke in SMACreER:Ai96 mouse under 2PLSM. Coupled with the real-time calcium oscillation changes trace of SMC before (dash line) and after (solid line) (occ.2hrep.22h) ischemic stroke.

File name: Supplementary movie 4

Description: Time-lapse imaging of primary-culture SMCs before and after CCCP treatment in the control virus and ME-Linker virus groups. The calcium signal was indicated by calcium indicator YTnC2-5.

File name: Supplementary movie 5

Description: Time-lapse imaging of calcium oscillation changes in SMC before and after (occ.2hrep.22h) ischemic stroke in SMACreER:ME-Linker mouse under 2PLSM. Coupled with the real-time calcium oscillation changes trace of SMC before (dash line) and after (solid line) (occ.2hrep.22h) ischemic stroke.

File name: Supplementary movie 6

Description: Time-lapse imaging of MCA before and after (occ.2hrep.22h) ischemic stroke in SMACreER:ME-Linker mice mouse under 2PLSM. Coupled with the real-time radius changes trace before (dash line) and after (solid line) (occ.2hrep.22h) ischemic stroke
