# Supplementary material for: Ca2+ oscillation in vascular smooth muscle cells control myogenic spontaneous vasomotion and counteract post-ischemic no-reflow
Source: Commun Biol. 2024 Mar 15;7:332. doi: 10.1038/s42003-024-06010-1 (PMC10942987; doi:10.1038/s42003-024-06010-1)
Supplement: Supplementary file 11 — Reporting Summary [file 42003_2024_6010_MOESM11_ESM.pdf]

nature portfolio

Corresponding author(s): Jinze Li, Jie-Min Jia

Last updated by author(s): Feb 21, 2024

Reporting Summary

Nature Portfolio wishes to improve the reproducibility of the work that we publish. This form provides structure for consistency and transparency in reporting. For further information on Nature Portfolio policies, see our Editorial Policies and the Editorial Policy Checklist.

Statistics

For all statistical analyses, confirm that the following items are present in the figure legend, table legend, main text, or Methods section.

n/a	Confirmed	
☐	☒	The exact sample size (n) for each experimental group/condition, given as a discrete number and unit of measurement
☐	☒	A statement on whether measurements were taken from distinct samples or whether the same sample was measured repeatedly
☐	☒	The statistical test(s) used AND whether they are one- or two-sided<br>*Only common tests should be described solely by name; describe more complex techniques in the Methods section.*
☒	☐	A description of all covariates tested
☒	☐	A description of any assumptions or corrections, such as tests of normality and adjustment for multiple comparisons
☐	☒	A full description of the statistical parameters including central tendency (e.g. means) or other basic estimates (e.g. regression coefficient) AND variation (e.g. standard deviation) or associated estimates of uncertainty (e.g. confidence intervals)
☐	☒	For null hypothesis testing, the test statistic (e.g. F, t, r) with confidence intervals, effect sizes, degrees of freedom and P value noted<br>*Give P values as exact values whenever suitable.*
☒	☐	For Bayesian analysis, information on the choice of priors and Markov chain Monte Carlo settings
☒	☐	For hierarchical and complex designs, identification of the appropriate level for tests and full reporting of outcomes
☐	☒	Estimates of effect sizes (e.g. Cohen's d, Pearson's r), indicating how they were calculated

Our web collection on statistics for biologists contains articles on many of the points above.

Software and code

Policy information about availability of computer code

Data collection	Mouse heart function was checked by transthoracic echocardiography (Vevo 3100, Visual Sonics). The blood pressure and heartrate of mouse were detected and analyzed using a noninvasive blood pressure measurement system (Kent, CODA), by cooperating with the physiological signal analytical system (Biopac, MP160). The body temperature of mouse was recorded by rectal thermometer (WANCE, TH-212). Flowmetry measurements in CBF were performed with the moorVMS-LDF monitor (Moor Instruments) equiped with a 785 nm laser. Blood flow in the mouse brain was recorded in real-time using an RFLSI III device (RWD Life Sciences, Shenzhen, China). Mice were live-imaged using a two-photon laser scanning microscope (Olympus, FLUOVIEW, FVMPE-RS) equipped with a cooled high-sensitivity GaAsP PMT detector and an ultrafast IR pulsed laser system (Spectra-Physics, InSight X3). TEM examination and image acquisition were conducted on a Talos L120C transmission electron microscope (Thermo Scientific, USA) equipped with a Ceta 16MP CMOS camera and Velox software (Thermo Scientific, USA). Live cells were imaged with a DeltaVision Ultra automated widefield microscope (GE Healthcare). Brain slices was imaged with a Zeiss Axio Zoom. V16 microscopy under bright field illumination.
Data analysis	Echocariograghy data with blinded genotypes were analyzed in Vevo Lab software (v5.5.1, FUJIFILM Sonosite Inc.). LDF data were alalyzed with the moorVMS-PC software (V3.1, Moor Instruments). Images acquired by Laser speckle Contrast Imaging device were processed by LSCI_V5.0. The numerical data concealed in the raw digital images were extracted and run on the software of Fiji (version 2.3.0/1.53f) or MATLAB (version R2021a). All statistical analyses and graphical illustrations were performed using GraphPad Prism 8 software (version 8.3.1, California, USA).

For manuscripts utilizing custom algorithms or software that are central to the research but not yet described in published literature, software must be made available to editors and reviewers. We strongly encourage code deposition in a community repository (e.g. GitHub). See the Nature Portfolio guidelines for submitting code & software for further information.

Data

Policy information about availability of data

All manuscripts must include a data availability statement. This statement should provide the following information, where applicable:

- Accession codes, unique identifiers, or web links for publicly available datasets
- A description of any restrictions on data availability
- For clinical datasets or third party data, please ensure that the statement adheres to our policy

All the data supporting the study are available within the article and its Supplementary Information files.

Research involving human participants, their data, or biological material

Policy information about studies with human participants or human data. See also policy information about sex, gender (identity/presentation), and sexual orientation and race, ethnicity and racism.

Reporting on sex and gender	n/a
Reporting on race, ethnicity, or other socially relevant groupings	n/a
Population characteristics	n/a
Recruitment	n/a
Ethics oversight	n/a

Note that full information on the approval of the study protocol must also be provided in the manuscript.

Field-specific reporting

Please select the one below that is the best fit for your research. If you are not sure, read the appropriate sections before making your selection.

☒ Life sciences ☐ Behavioural & social sciences ☐ Ecological, evolutionary & environmental sciences

For a reference copy of the document with all sections, see nature.com/documents/nr-reporting-summary-flat.pdf

Life sciences study design

All studies must disclose on these points even when the disclosure is negative.

Sample size	No statistical method was utilized to calculate the sample size. The sample size was determined based on the literature (Neuron. 2015 Jul 1;87(1):95-110.; Cell Metab. 2020 Apr 7;31(4):791-808.e8.) and our previous studies (Nat Methods. 2017 Feb;14(2):160-166.; Commun Biol. 2022 Feb 16;5(1):136.). 3R (reduction, replacement, refinement) principle was also considered in our experiment design. The sample size and the number of biological repeats are provided in the relevant figure legends and methods.
Data exclusions	During 2PLSM live imaging, mice with unsuccessful cranial window (obscure window due to the chronic bleeding of the vessels in the cranial bone) surgery were excluded (overall 2 mice in this project). In LSCI assays, the ipsilateral relative CBV were less than 20% comparing to contralateral in all mice during occlusion, indicating the successful of the MCAO surgery, and no mice were excluded. In paired analysis (Fig.1f,1g,1h,3c,3f,3g,3h,4k,4l), all the paired data collected in the identical location with two time points (before and occ.2hrep.22h) were used, unpaired data (source data from only one time point either before or occ.2hrep.22h) were excluded.
Replication	Each experiment presented in the study was repeated in multiple separate experiments or multiple animals (between 3- 20). All results in the paper are drawn from the analysis of multiple repeats and animals.
Randomization	Animals were assigned randomly to experimental and control groups.
Blinding	Investigators were blinded to sample identity during quantitative analysis.

Reporting for specific materials, systems and methods

We require information from authors about some types of materials, experimental systems and methods used in many studies. Here, indicate whether each material, system or method listed is relevant to your study. If you are not sure if a list item applies to your research, read the appropriate section before selecting a response.

Materials & experimental systems

n/a	Involved in the study
☐	☒ Antibodies
☒	☐ Eukaryotic cell lines
☒	☐ Palaeontology and archaeology
☐	☒ Animals and other organisms
☒	☐ Clinical data
☒	☐ Dual use research of concern
☒	☐ Plants

Methods

n/a	Involved in the study
☒	☐ ChIP-seq
☒	☐ Flow cytometry
☒	☐ MRI-based neuroimaging

Antibodies

Antibodies used	Map2 (Abclonal, Cat# A22205, Lot#6100000580) and NenN (Abclonal, Cat #A19086, Lot#4000003560) antibody were used for histological neurological injury assessment in this study.
Validation	The validation of Map2 (Abclonal, Cat# A22205) antibody can be found at manufacturer's website https://abclonal.com.cn/catalog/A22205; For NenN (Abclonal, Cat #A19086) antibody, related information can be found at manufacturer's website https://abclonal.com.cn/catalog/A19086.

Animals and other research organisms

Policy information about studies involving animals; ARRIVE guidelines recommended for reporting animal research, and Sex and Gender in Research

Laboratory animals	The following mouse strains were used: wild type (C57BL/6J), SMACreER (Wendling et al., 2009, genesis 47, 14–18), Cdh5CreER (Blood. 2009 May 28;113(22):5680-8.), Ai14 (JAX:007914), Ai47 (Daigle et al., 2018, Cell 174, 465–480), Ai96 (JAX:028866), and RCL-ME-Linker (this paper, see methods). 3 to 6 months old adult mice were used in this study. All mice were bred and maintained in a specific-pathogen-free animal room on a 12-hour light-dark cycle and provided food and water ad libitum.
Wild animals	No wild animals were involved in this study.
Reporting on sex	Both male and female mice were used in this study. There is no significant different in arteriolar vasomotion index between male and female mice, indicating sex-related factors had very limited impact on the characteristics of arteriolar myogenic vasomotion physiologically. See supplemental figure 2 and the corresponding discussion in the main text in this study.
Field-collected samples	No field-collected samples were involved in this study.
Ethics oversight	All animal protocols were approved by the Institutional Animal Care and Use Committee (IACUC) at our (anonymmous during DBPR) University.

Note that full information on the approval of the study protocol must also be provided in the manuscript.
